# Supplementary material for: Bayesian model and selection signature analyses reveal risk factors for canine atopic dermatitis
Source: Commun Biol. 2022 Dec 8;5:1348. doi: 10.1038/s42003-022-04279-8 (PMC9731970; doi:10.1038/s42003-022-04279-8)
Supplement: Supplementary file 2 — Supplementary information [file 42003_2022_4279_MOESM2_ESM.pdf]

Supplementary Information

Supplementary Figures

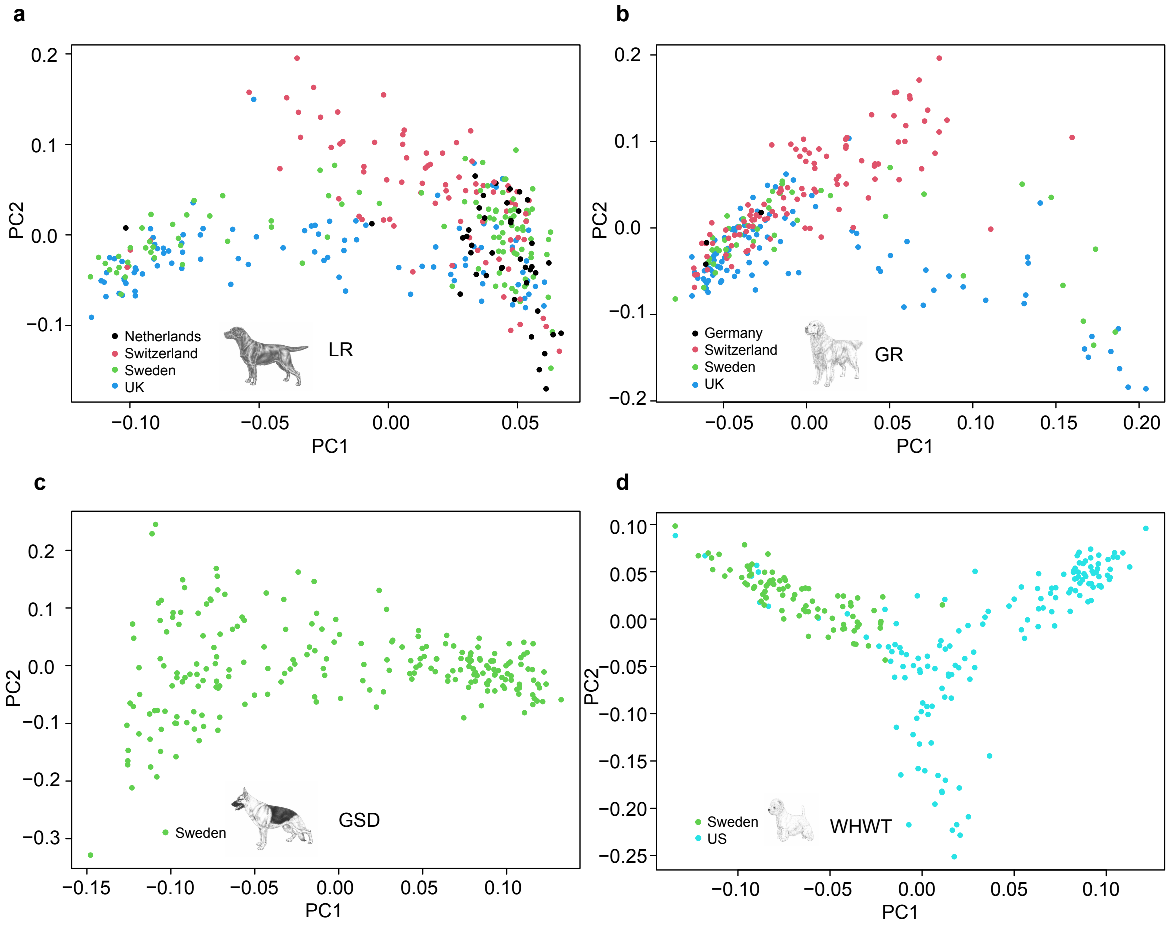

Supplementary Figure 1 PCA plots displaying the genetic relationship color coded with country of origin for each breed, LR (**a**), GR (**b**), GSD (**c**), and WHWT (**d**).

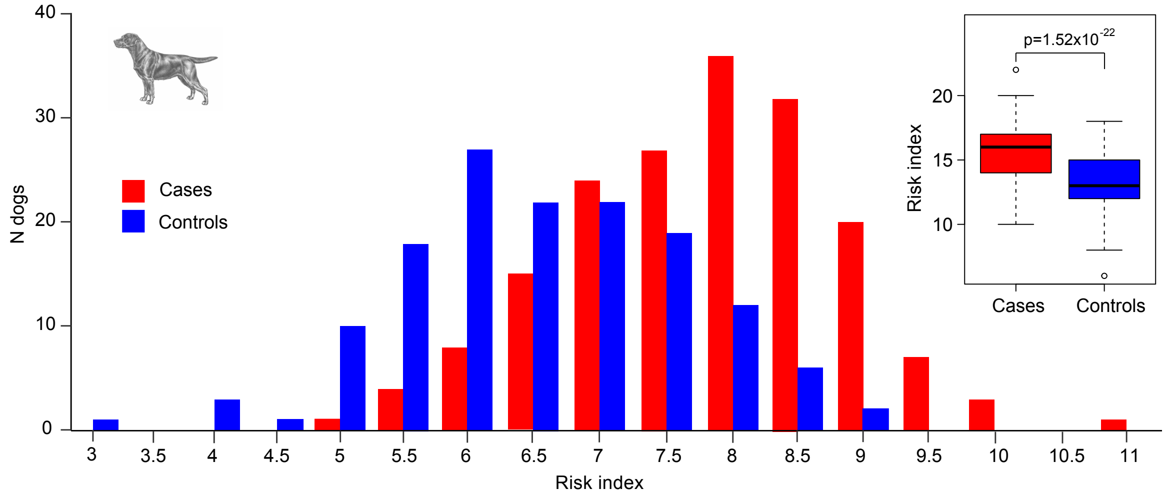

Supplementary Figure 2 Whole genome selection analysis presenting XP-EHH values from comparing from comparing the case with the control populations of LR (**a**), GR (**b**), GSD (**c**), and WHWT (**d**).

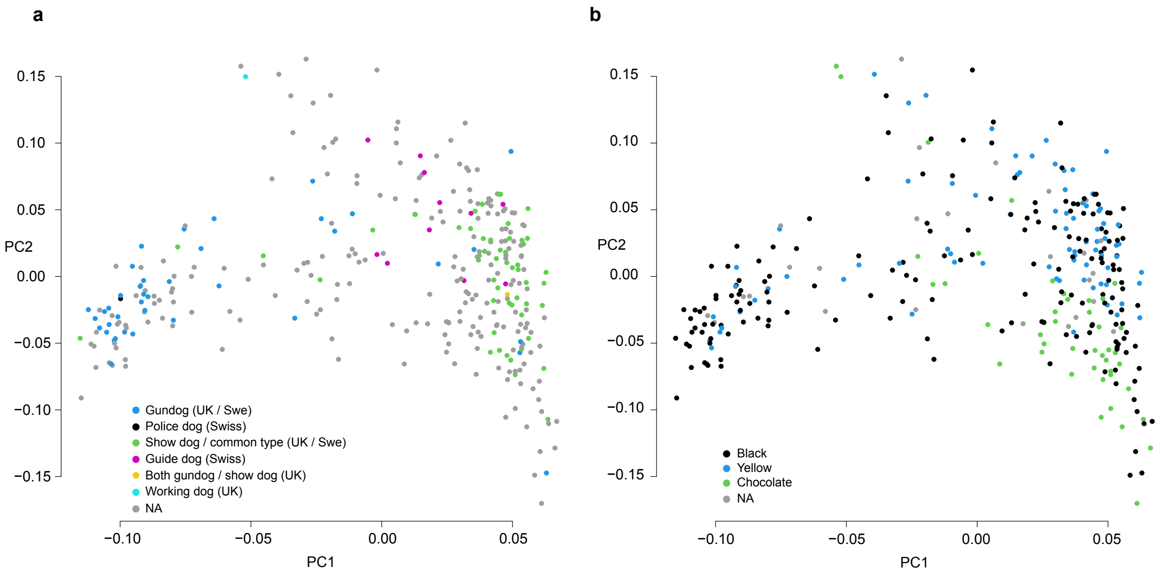

Supplementary Figure 3 LR from Swedish kennels that were listed as gundog type kennels are marked blue in the relationship matrix. The list of gundog type kennels included data from kennels with puppies born 2006-2021. (Three Swedish LR in the low PC1 cluster were called as common types but these are from gundog kennels active before 2006, i.e., not included in the current gundog kennel list.) Furthermore, seven UK LR were defined as gundogs (blue), two as show dogs (green), one was both gundog / show dog, and one working dog (turquoise) based on owner questionnaires. One police dog (black) and eleven guide dogs for the blind (purple) were defined in the Swiss cohort (**a**). The coat color was extracted from all dogs except 26 showing that the chocolate color (green) does not exist in the gundog cluster, as expected (**b**).

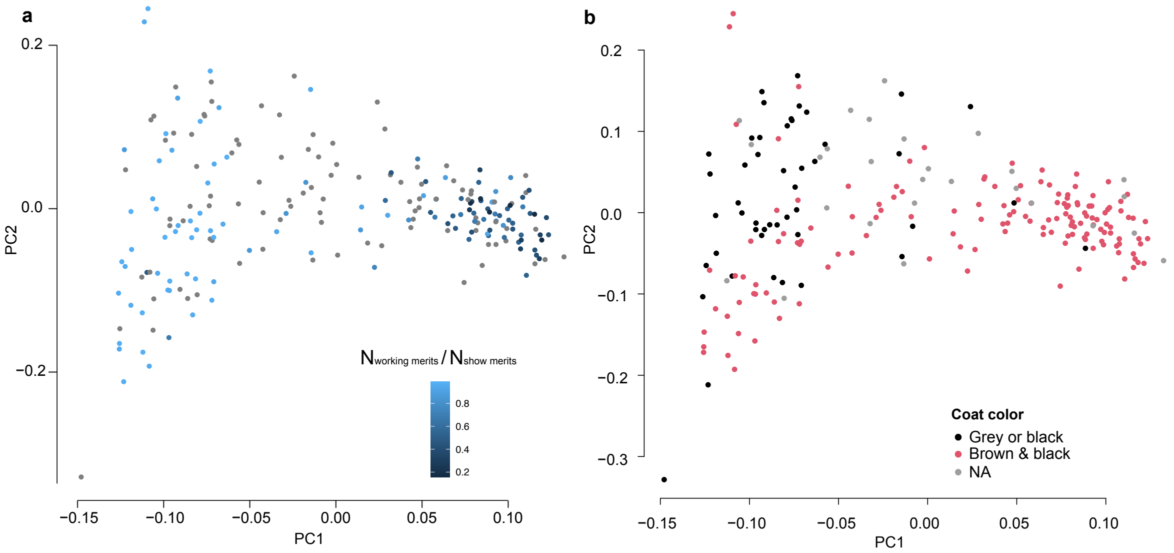

Supplementary Figure 4 Out of the 219 GSD included in our analysis, 121 have kennel names (the rest are marked in grey) and the dog's kennel's proportion of working merits (N) over number of show merits (N) is reflected by the color scale ranging from light blue (higher proportion of working merits) to dark blue (higher proportion of show merits) in the PCA plot (**a**). Coat color were available from 192 GSDs and grouping colors into two classes; 1) grey or black, 2) brown and black, shows that grey or black color is more common in the low PC1 cluster (**b**).

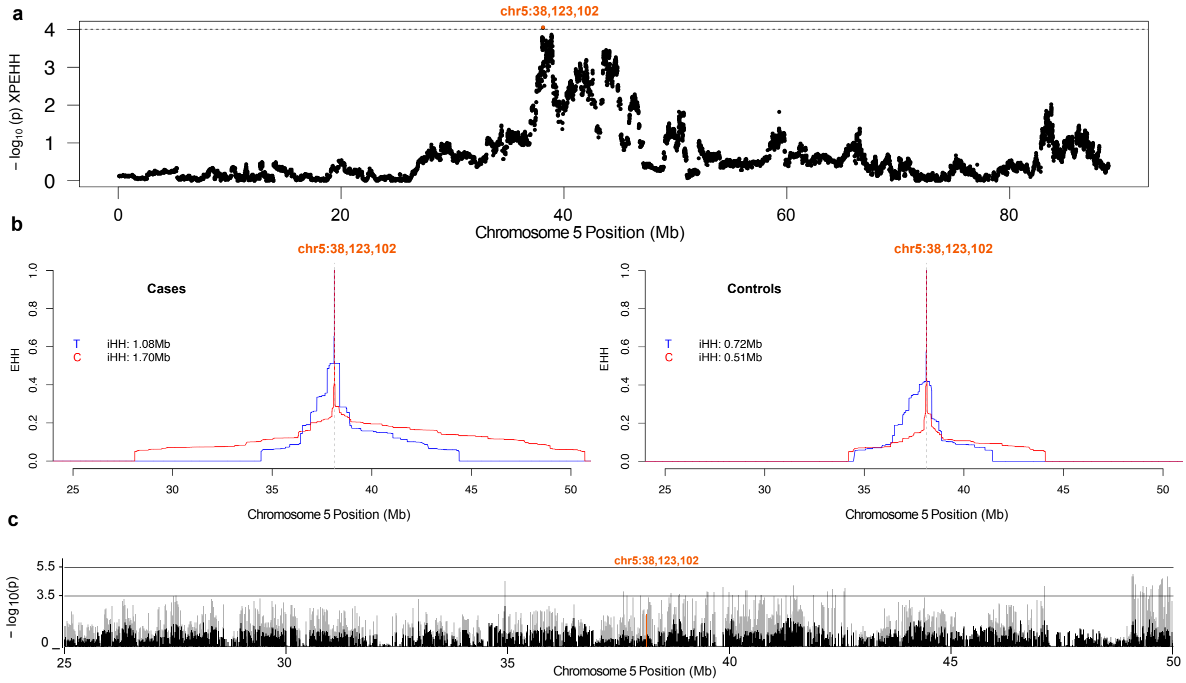

Supplementary Figure 5 A candidate region of selection was detected around 38Mb on chromosome 5 in LR (**a**). EHH plots for the top selection variant chr5:38,123,102 are presented for cases and controls (**b**), and the association with canine AD (--assoc in grey and --logistic in black) is shown across the locus (**c**).

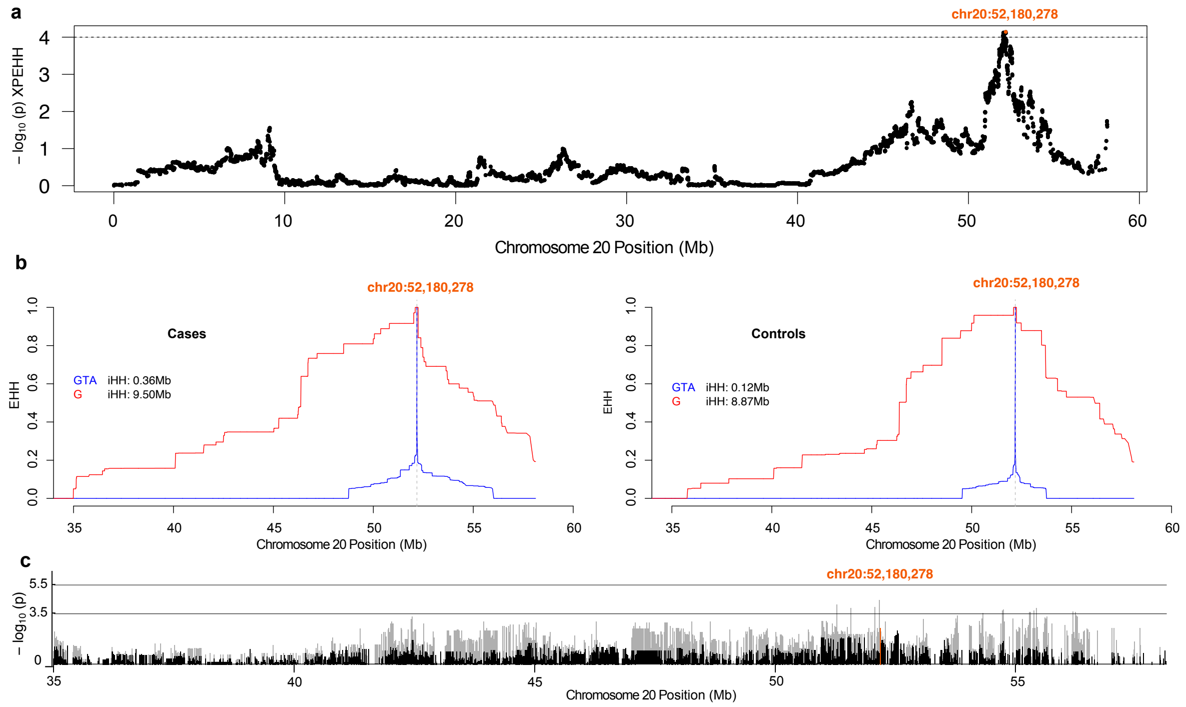

Supplementary Figure 6 A candidate region of selection was detected around 52Mb on chromosome 20 in GSD (**a**). EHH plots for the top selection variant chr5:38,123,102 are presented for cases and controls (**b**), and association with canine AD (--assoc in grey and --logistic in black) is shown across the locus (**c**).

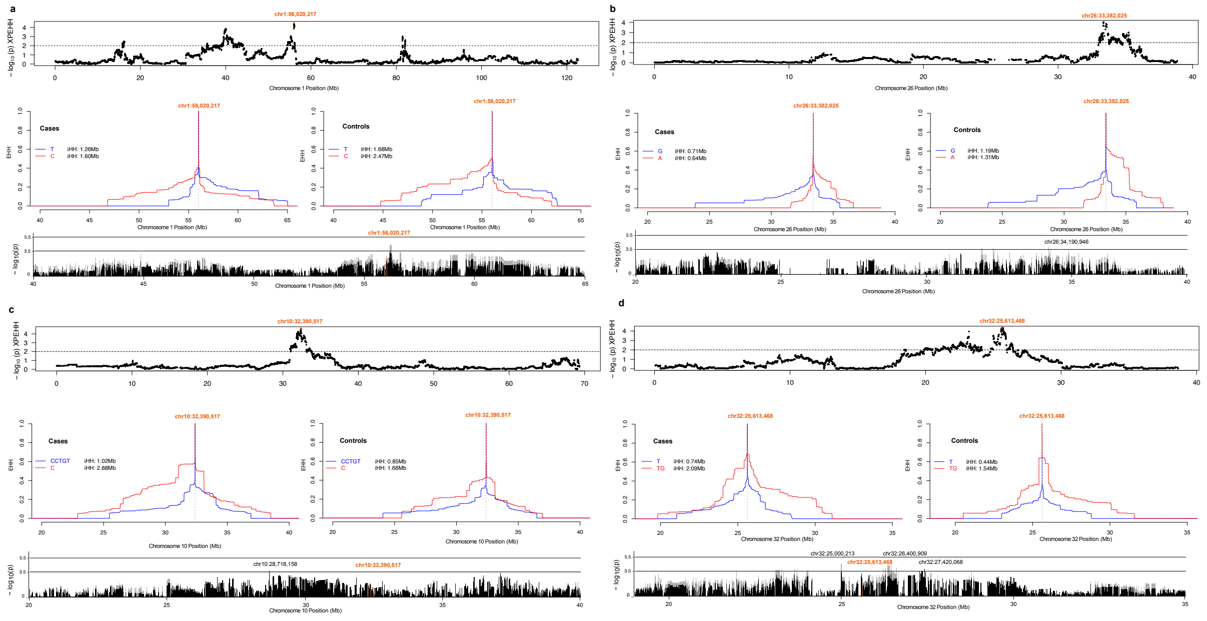

Supplementary Figure 7 Selection loci in on chromosomes 1 (**a**), 10 (**b**), 26 (**c**), and 32 (**d**) in WHWT. Panels show, from top down, selection signals, EHH plots for cases and controls, and association with canine AD (--assoc in grey and --logistic in black) across the selection loci. The top selection variants are marked in orange.

Simple Tandem Repeat Information

Period: 121
Copies: 2.0

Consensus size: 121

Match Percentage: 96%

Insert/Delete Percentage: 1%

Score: 454

Entropy: 1.920

Sequence:
AGAGTATCTTTTTCGCACCATAGAGGCCACCCTACGGAAAGAAGGCAGAGCACTGAGGAGTAAAAAAGAACTAGAAGAGACAGACTTTAAAAA
CTTAGAACTAGGGTGGCTTAGTTGATTA

Position: chr17:60228995-60229238

Genomic Size: 244

Supplementary Figure 8 The Illumina CanineHD SNP BICF2G630286126 was initially defined as the top effect variant on chromosome 17 but was located in a simple tandem repeat, which prompted further evaluation.

Tandem copy 1=A, Tandem copy 2=G

BICF2G630286126

```
AGAGTATCTTTTTCGCACCATAGAGGCCACCCTACGGAAAGAAGGTAGAGCACTGAGGAGTAAAAAAGAACTAGAAGAGACAGACTTTAAAAACTTAGAACT
AGGGTGGCTTAGTTGATTAAGAGTATCTGTTTTGCACCATGGAGGCCACCCTACGGAAAGAAGGCAGAGCACTGAGGAGTAAAAAAGAACTAGAAGAGACAG
ACTTTAAAAACTTAGAACTAGGGTGGCTTAGTTGATTA
```

BICF2G630286126

```
STR_c1           1 AGAGTATCTTTTTCGCACCATAGAGGCCACCCTACGGAAAGAAGGTAGAG     50
                   |||||||||.|||.|||||||.|||||||||||||||||||||||.||||
STR_c2           1 AGAGTATCTGTTTTGCACCATGGAGGCCACCCTACGGAAAGAAGGCAGAG     50

STR_c1          51 CACTGAGGAGTAAAAAAGAACTAGAAGAGACAGACTTTAAAAACTTAGAA    100
                   ||||||||||||||||||||||||||||||||||||||||||||||||||
STR_c2          51 CACTGAGGAGTAAAAAAGAACTAGAAGAGACAGACTTTAAAAACTTAGAA    100

STR_c1         101 CTAGGGTGGCTTAGTTGATTA    121
                   |||||||||||||||||||||
STR_c2         101 CTAGGGTGGCTTAGTTGATTA    121
```

Dog	STR c1	STR c2
Tascha cf3	A	G
Mischka cf4	A	G
1295 ctrl	A	G
1299 ctrl	A	G
14427 case	A	G
14440 case	A	G

Supplementary Figure 9. The BICF2G630286126 has an A in the first copy of the STR and G in the second. From Sanger sequencing we confirmed that both cases and controls of LR were A/A for the first STR copy and G/G for the second. The same was the case for the reference genomes canFam3.1 (Tascha cf3) and canFam4 (Mischka cf4).

Supplementary Tables

Supplementary Table 1. Number of dogs from each country. Number of dogs before QC (N dogs in final datasets)

Contry	Status	LR	GR	GSD	WHWT
Sweden	cases	68 (66)	38 (31)	111 (106)	61 (55)
Sweden	controls	44 (36)	21 (15)	120 (113)	37 (31)
UK	cases	62 (54)	48 (42)	0	0
UK	controls	46 (43)	65 (61)	0	0
US	cases	0	0	0	97 (82)
US	controls	0	0	0	85 (67)
Switzerland*	cases	39 (38)	77 (68)	0	0
Switzerland*	controls	46 (42)	42 (36)	0	0
Netherlands	cases	20 (20)	0	0	0
Netherlands	controls	20 (20)	0	0	0
Germany	cases	0	2 (2)	0	0
Germany	controls	0	1 (1)	0	0
France	cases	0	0	0	0
France	controls	1 (1)	0	0	0
Finland	cases	0	0	0	0
Finland	controls	1 (1)	0	0	0
	Total cases	189 (178)	165 (143)	111 (106)	158 (137)
	Total controls	158 (143)	129 (113)	120 (113)	122 (98)
	Total	347 (321)	294 (256)	231 (219)	280 (235)

*Samples from the Netherlands, Germany, France, and Finland were included in the Swiss cohort.

Supplementary Table 2. Datasets passing quality control, LD-pruning, and relatedness filtering (N variants / N dogs)

	1. Imputed dataset	2. Imputed dataset after additional QC	3. Variants after LD-pruning	4. Imputed dataset after adding QC:ed genotyped SNPs (chrX excl.)	5. Related dogs excl.	6. Extra LD-pruning on final datasets	Genotyped dataset
	(--geno 0.05 --mind 0.05 --maf 0.05)	(--geno 0.02 --mind 0.05 --maf 0.05)	(--indep-pairwise 25 5 0.999)	(--maf 0.05 --geno 0.05 --mind 0.05)	(KING 0.177 from genotype data. --maf 0.05)	(--indep-pairwise 50 5 0.99) keeping all genotyped SNPs	(--geno 0.05 --mind 0.05 --maf 0.05 --not-chr 39)
**LR**	4,144,809 / 336	3,451,252 / 335	829,631	854,902 / 335	852,568 / 321	543,782 / 321	110,592
**GR**	3,791,168 / 287	3,378,987 / 287	673,407	721,148 / 287	718,580 / 256	397,687 / 256	98,470
**GSD**	3,472,380 / 229	3,000,396 / 229	541,925	590,027 / 229	587,270 / 219	NA	94,677
**WHWT**	3,445,719 / 277	3,011,398 / 276	532,475	581,361 / 276	576,547 / 235	NA	93,149

Supplementary Table 3. Phenotypic variance explained by AD-loci and risk index in LR

Chr	Variant	Estimate	Std.Error	t-value	Pr(>|t|)	Sign	Df	SumSq	Mean Sq	F-value	Pr(>F)	variance explained (%)	maf (cases)	maf (controls)
	(Intercept)	-1.22	0.16	-7.83	7.81E-14	***								
3	BICF2G630347384	0.43	0.09	4.6	6.20E-06	***	1	5.8	5.8	36.5	4.45E-09	7.3	0.07	0.206
4	BICF2S23727494	0.22	0.07	3.12	1.96E-03	**	1	3	3	18.7	2.10E-05	3.8	0.264	0.385
4	4_63451285	0.15	0.08	1.96	5.09E-02	.	1	1.8	1.8	11.2	9.03E-04	2.3	0.167	0.292
5	TIGRP2P74583	0.26	0.1	2.57	1.07E-02	*	1	1.4	1.4	8.6	3.62E-03	1.7	0.261	0.343
17	17_59225133	0.28	0.08	3.56	4.23E-04	***	1	1.3	1.3	8.3	4.27E-03	1.7	0.171	0.28
21	21_46702815	0.26	0.07	3.88	1.26E-04	***	1	4.9	4.9	31	5.65E-08	6.2	0.368	0.217
26	26_34371008	0.26	0.08	3.28	1.15E-03	**	1	3	3	18.9	1.85E-05	3.8	0.29	0.164
33	TIGRP2P388667	0.23	0.07	3.24	1.34E-03	**	1	2.9	2.9	18.3	2.56E-05	3.7	0.216	0.374
34	34_26660144	0.17	0.07	2.52	1.22E-02	*	1	0.2	0.2	1.6	2.12E-01	0.3	0.393	0.482
36	36_24333961	0.27	0.09	2.96	3.36E-03	**	1	1.5	1.5	9.1	2.72E-03	1.8	0.077	0.171
37	37_27652421	0.12	0.07	1.67	9.68E-02	.	1	0.2	0.2	1.2	2.83E-01	0.2	0.198	0.257
	PC1	2.37	0.47	5.06	7.41E-07	***	1	4.1	4.1	25.6	7.16E-07	5.2		
	PC2	-0.48	0.45	-1.08	2.82E-01		1	0.2	0.2	1.2	2.82E-01	0.2		
	Residuals						307	49	0.2	NA	NA	61.8		
		**Estimate**	**Std.Error**	**t-value**	**Pr(>|t|)**	**Sign**	**Df**	**SumSq**	**Mean Sq**	**F-value**	**Pr(>F)**	**variance explained (%)**		
	(Intercept)	-1.08	0.14	-8	2.34E-14	***								
	risk index	0.23	0.02	12.26	1.56E-28	***	1	21	20.9	131.3	1.14E-25	26.4		
	PC1	2.71	0.4	6.82	4.53E-11	***	1	7.4	7.4	46.5	4.64E-11	9.3		
	PC2	-0.72	0.4	-1.78	7.65E-02	.	1	0.5	0.5	3.2	7.65E-02	0.6	-	
	Residuals						317	51	0.2	NA	NA	63.7		

Supplementary Table 4. Phenotypic variance explained by AD-associated loci in GR, GSD, and WHWT, and the risk index in WHWT.

	Estimate	Std.Error	t-value	Pr(>|t|)	Sign	Df	SumSq	MeanSq	F-value	Pr(>F)	variance explained (%)
**GR**											
(Intercept)	1.45	0.04	40.02	6.00E-111	***						
23:8319756	0.41	0.09	4.64	5.70E-06	***	1	1.47	1.47	7.4	6.97E-03	2.3
PC1	2.27	0.44	5.2	4.20E-07	***	1	5.16	5.16	25.98	6.79E-07	8.2
PC2	0.66	0.44	1.51	0.13		1	0.4	0.4	2.01	1.58E-01	0.6
PC3	-2.48	0.44	-5.61	5.40E-08	***	1	6.25	6.25	31.45	5.38E-08	9.9
Residuals						251	49.85	0.20	NA	NA	79
**GSD**											
(Intercept)	0.68	0.08	8.29	1.32E-14	***						
9:15534865	0.5	0.11	4.71	4.53E-06	***	1	1.27	1.27	10.08	1.72E-03	2.32
logIgA	0.45	0.09	5.05	9.54E-07	***	1	14.14	14.14	112.25	2.53E-21	25.85
logAge	0.81	0.12	6.98	3.70E-11	***	1	7.28	7.28	57.81	9.22E-13	13.31
PC1	1.7	0.31	5.43	1.53E-07	***	1	3.85	3.85	30.58	9.39E-08	7.04
PC2	0.11	0.33	0.33	0.74		1	0.04	0.04	0.29	5.91E-01	0.07
PC3	-1.17	0.35	-3.36	0.000921	***	1	1.42	1.42	11.3	9.21E-04	2.6
Residuals						212	26.7	0.13	NA	NA	48.81
**WHWT**											
(Intercept)	1.19078	0.06559	18.154	7.93E-46	***						
10:8565303	0.38	0.09	4.24	3.23E-05	***	1	4.02	4.02	20.15	1.15E-05	7.3
15:55988962	0.41	0.08	4.86	2.21E-06	***	1	4.95	4.95	24.81	1.27E-06	8.9
PC1	0.8	0.47	1.71	0.09	.	1	0.62	0.62	3.1	7.98E-02	1.1
PC2	1.16	0.45	2.59	0.01	*	1	1.34	1.34	6.71	1.02E-02	2.4
						223	44.49	0.2	NA	NA	80.3
**WHWT**											
(Intercept)	1.19	0.06	18.47	6.73E-47	***						
risk index (two loci)	0.4	0.06	6.79	9.84E-11	***	1	8.92	8.92	44.89	1.66E-10	16.1
PC1	0.82	0.45	1.82	0.07	.	1	0.67	0.67	3.37	6.79E-02	1.2
PC2	1.15	0.45	2.59	0.01	*	1	1.33	1.33	6.7	1.03E-02	2.4
						224	44.5	0.2	NA	NA	80.3

Footnote: Fixed effects were defined by “fitNullModel " in GENESIS to have significant ($p<0.05$) effect on the trait.

Supplementary Table 5. Phased core haplotypes in the two sequenced LR cases

			Case #1		Case #2	
Name	cf4	pos	H1	H2	H1	H2
chr17:a	chr17	58344321	C	T	C	T
chr17:b	chr17	58637142	c	c	c	c
chr17:c	chr17	58742834	A	C	C	A
chr17:d	chr17	58808329	A	G	A	G
chr17:e	chr17	59787164	C	T	C	T
chr17:f	chr17	59881693	(G)-	(G)ACTC	(G)ACTC	(G)-
chr17:g	chr17	60101803	C	T	T	C
chr17:h	chr17	60103367	T	C	C	T
chr17:i	chr17	60347720	T	T	A	T

core haplotype RISK ALLELE

Supplementary Table 6. Variants in candidate regions under selection with phyloP >2.56

Chr	canFam3.1: pos	canFam4:pos	XP-EHH	log10(p)	closest gene(s)	genomic position	phyloP	note
3	74,063,361	74,636,332	4.4	5.1	*KLF3/TBC1D1, TBC1D1**	intergenic /intronic*	2.6	in GH04J038153
3	74,165,646	74,739,019	4.2	4.6	*TBC1D1*	intronic	4.3	in GH04J038049
3	74,185,575	74,758,672	4.2	4.6	*TBC1D1, TBC1D1* and BCCIP**	intronic. intronic*	5.0	exonic BCCCIP
10	32,378,918	33,432,268	4.1	4.4	*TCP11L2/CKAP4, TCP11L2**	intergenic. intronic*	3.1	in ENCODE Candidate Cis-Regulatory Elements (cCREs) combined from all cell types (EH38E1640927)
19	42,562,348	44,063,366	-4.1	4.4	*LRP1B, HADH.7/LRP1B**	intronic. intergenic*	3.4	no marks over this variant
19	43,825,832	45,468,548	-4.3	4.7	*LRP1B/KYNU, LRP1B**	intergenic. intronic*	3.4	in stat5 tfbs
19	43,904,161	45,546,862	-4.4	5	*LRP1B/KYNU, LRP1B**	intergenic. intronic*	4.7	exonic TLE4. overlapping LHX3 tfbs
19	43,911,822	45,554,517	-4.5	5.1	*LRP1B/KYNU, LRP1B**	intergenic. intronic*	3.7	several tfbs
19	43,989,619	45,632,167	-4.5	5.1	*LRP1B/KYNU, LRP1B**	intergenic. intronic*	2.9	GRLH2. Gflib tfbs
19	44,090,617	45,733,226	-4.6	5.3	*LRP1B*	exonic	7.0	LRP1B exon. several tfbs
19	44,374,782	46,016,415	-4.3	4.8	*LRP1B/KYNU, LRP1B**	intergenic. intronic*	5.0	NR6A1. THAP tfbs. in ENCODE Candidate Cis-Regulatory Elements (cCREs) combined from all cell types (EH38E2037114)
32	25,720,434	14,167,098	3.9	4.1	*TET2/CXXC4*	intergenic	3.7	on the very edge of tfbs

* canFam4 position (if different from canFam3.1)

Supplementary Table 7. Results from selection genes in STRING (*Homo sapiens*)

**#category**	**term ID**	**term description**	**observed gene count**	**background gene count**	**strength**	**false discovery rate**	**matching proteins in your network (labels)**	**matching proteins in your network (IDs)**
TISSUES	BTO:0000740	Myeloid leukemia cell line	5	28	1.41	0.0056	*KLF3, FCER2, MCOLN1, MLLT4, PRAM1*	9606.ENSP00000261438.9606.ENSP00000264072.9606.ENSP00000264079.9606.ENSP00000375960.9606.ENSP00000408342
TISSUES	BTO:0001883	Acute myeloid leukemia cell line	4	21	1.44	0.0234	*KLF3, MCOLN1, MLLT4, PRAM1*	9606.ENSP00000261438.9606.ENSP00000264079.9606.ENSP00000375960.9606.ENSP00000408342
TISSUES	BTO:0000737	Leukemia cell line	6	85	1.01	0.0253	*KLF3 (chr3), FCER2 (chr20), MCOLN1 (chr20), RPS6 (chr5), MLLT4 (alias for AFDN chr1), PRAM1 (chr20)*	9606.ENSP00000261438.9606.ENSP00000264072.9606.ENSP00000264079.9606.ENSP00000369757.9606.ENSP00000375960.9606.ENSP00000408342

Supplementary Table 8. Results from XP-EHH genes in STRING (*Canis lupus familiaris*)

#category	GO Component	STRING clusters	STRING clusters	STRING clusters	STRING clusters
term ID	GO:0045335	CL:35647	CL:31523	CL:35629	CL:31467
term description	Phagocytic vesicle	Mixed, incl. positive regulation of humoral immune response mediated by circulating immunoglobulin, and dsrna transport	Mixed. incl. regulation of brood size, and ccr5 chemokine receptor binding	Mostly uncharacterized, incl. dsrna transport, and protein antigen binding	Mostly uncharacterized, incl. regulation of brood size, and circadian rhythm - plant
observed gene count	6	4	3	5	4
background gene count	87	5	5	45	33
strength	1.04	2.11	1.98	1.25	1.29
false discovery rate	0.0373	0.00075	0.0165	0.0165	0.0493
matching proteins in your network (labels)	*APPL2, TLR6, RAB11A, STXBP2, TLR1, RAB11B*	*PCP2, MCEMP1, FCER2, PET100*	*TEKT3, CDRT4, TVP23B*	*PCP2, ZFP2, MCEMP1, FCER2, PET100*	*RFX4, TEKT3, CDRT4, TVP23B*
Genes (chr)	*APPL2 (chr10)*	*PCP2 (chr20)*	*TEKT3 (chr5)*	*PCP2 (chr20)*	*RFX4 (chr10)*
	TLR6 (chr3)	*MCEMP1 (chr20)*	*CDRT4 (chr5)*	*ZFP2 (chr20)*	*TEKT3 (chr5)*
	RAB11A (chr20)	*FCER2 (chr20)*	*TVP23B (chr5)*	*MCEMP1 (chr20)*	*CDRT4 (chr5)*
	STXBP2 (chr20)	*PET100 (chr20)*		*FCER2 (chr20)*	*TVP23B (chr5)*
	TLR1 (chr3)			*PET100 (chr20)*	
	RAB11B (chr20)				

Supplementary Table 9. Term "connective tissue" from STRING (*Homo Sapiens*): summary of genes represented by BayesR and XP-EHH regions

**Gene**	**Chr (BayesR region)**	**Chr (XP-EHH region)**	**Breed**
HMGA2	chr10		WHWT
CTSK	chr17		LR
GPR89A	chr17		
VPS72	chr17		
CTSS	chr17		
ECM1	chr17		
C1orf54	chr17		
ITGA10	chr17		
SELENBP1	chr17		
ANP32E	chr17		
RPSA	chr23		GR
NACA	chr23		
SLC2A9	chr3		LR
WDR1	chr3		
ATP6V1E1	chr34		LR
CD74	chr4		LR
SPARC	chr4		
NDST1	chr4		
ANXA6	chr4		
G3BP1	chr4		
GSTP1	chr4		
CALB2	chr5		LR
IL34	chr5		
IST1	chr5		
THBS2		chr1	WHWT
CRY1		chr10	WHWT
ALDH1L2		chr10	
MTERF2		chr10	
FSTL1 (alias OCC1)		chr10	
KYNU		chr19	GSD
RETN		chr20	GSD
ANGPTL4		chr20	
INSR		chr20	
PPA2		chr32	WHWT
TET2		chr32	
RPS6		chr5	LR
PMP22		chr5	
Total	7 bayesR regions	6 selection regions	

Supplementary Note 1

Exclusion of chromosome 17 SNP chr17:b from further evaluation
canFam3.1 chr17:57,977,924-57,977,925, canFam4 chr17:58,637,141-58,637,142, (referred to as chr17:b) is situated inside a LINE repeat with the indicated alleles from the imputed data as (C)AG and (C). In the Chromium Dogs dataset there is a variant annotated at canFam4: chr17:58,637,143-58,637,144 (C)AG and (C). When we inspect these genomic positions in the Nanopore sequenced data in IGV, we conclude that the controls have a variant at canFam4 chr17: 58,637,142-58,637,143, following the indicated alleles from the Chromium Dogs and the imputed dataset (C)AG and (C), while the cases have a variant at canFam3.1: chr17: 57,977,925-57,977,926, canFam4: chr17: 58,637,144-58,637,145 (A)GG and (A). Positions canFam4: chr17: 58,637,143-58,637,144 do not lift to canFam3.1. Since this variant is located in a LINE and show differing alleles and positions in the sequenced cases and controls, we excluded it from further evaluation.

Exclusion of chromosome 17 SNP BICF2G630286126 from analysis

SNP BICF2G630286126 from Illumina CanineHD (173,662 SNPs) genotyping BeadChip was indicated one of the top ranked variants on chromosome 17 from the initial BayesR analysis, with genotypes A/A in controls and A/G in cases. As a result of that, we aimed to verify the position in the Nanopore reads from the two controls and two cases sequenced, and discovered no variation among them. We then submitted the four individuals for Sanger sequencing of this region, with identical outcome, i.e., no variation at the position. BICF2G630286126 is located in a Simple Tandem Repeat (STR) on canFam3.1 chr17:60,228,995-60,229,238 (**Supplementary Figure 8**). This STR consists of two copies, identical to 96.1%. One of the four positions differing between them is the position for BICF2G630286126, with A in the first copy of the STR and G in the second. The two cases and two controls showed to all be A/A for the first copy and G/G for the second, which is also the case for the reference genomes canFam3.1 (Tascha cf3) and canFam4 (Mischka cf4; **Supplementary Figure 9**). Therefore, was the SNP BICF2G630286126 excluded from analysis.

PCR and Sanger sequencing for evaluation of chromosome 17 BICF2G630286126
DNA from LR sequenced with ONT (ID1, ID2, ID3, ID4) was amplified with primers F: 5'-GCCTGAAATGCCTGGTGTAG-3' and R:5'-AAAATCCCAAGCAGACTGGC-3' producing a PCR product covering BICF2G630286126. With reagents from the kit AmpliTaq Gold™ DNA Polymerase with Buffer II and $MgCl_2$ (Applied Biosystems, Thermo Scientific) the reaction was mixed as follows in a total volume of 20 µl: 1X Buffer II, 1.5 mM $MgCl_2$, 20 µM dNTPs, 0.7 U AmpliTaq Gold, 100 nM of F and R primer each, 30 ng's of DNA and dH_2O up to 20 µl total volume. PCR reactions were amplified with PCR program 94°C 10 min, followed by 6 cycles of 94°C 30s, 61-56°C 30s, 72°C 30s, 34 cycles of 94°C 30s, 55°C 30s, 72°C 30s, and ended with 72°C 5 min. 5 µl of the PCR products were run on a 1% agarose gel and the remaining reaction was cleaned up with in a total volume of 17 µl: 10 U of Exonuclease (EN0582, Thermo Fisher), 0.5 U of FastAP Thermosensitive Alkaline Phosphatase (EF0651, Thermo Fisher), 1 ul Buffer FastAP (Thermo Fisher) and 5 ul PCR product. The reaction was incubated at 30°C for 30 min followed by 85°C for 15 min. Reactions for Sanger sequencing were mixed as follows in a total volume of 17 µl: 3 µl of purified PCR product, 1.2 µM of F or R primer and dH_2O up to 17 µl. The sanger sequencing was performed by Eurofins Genomics and sequences were analyzed in Codon Code Aligner v10.0.1.
