# Supplementary material for: Bayesian model and selection signature analyses reveal risk factors for canine atopic dermatitis
Source: Commun Biol. 2022 Dec 8;5:1348. doi: 10.1038/s42003-022-04279-8 (PMC9731970; doi:10.1038/s42003-022-04279-8)
Supplement: Supplementary file 3 — Description of Additional Supplementary Files [file 42003_2022_4279_MOESM3_ESM.docx]

**Description of Additional Supplementary Files**

File name: Supplementary Data 1

Description: Canine AD-effect variants from BayesR analyses

File name: Supplementary Data 2

Description: Source data to Figure 2

File name: Supplementary Data 3

Description: All variants in LD with any of the effect variants in non-LD pruned imputed dataset

File name: Supplementary Data 4

Description: Novel variants identified in the four sequenced LRs

File name: Supplementary Data 5

Description: SVs identified in the two sequenced LR cases

File name: Supplementary Data 6

Description: Homozygous blocks identified in the Nanopore sequenced LRs

File name: Supplementary Data 7

Description: Variants passing the threshold of XP-EHH -log10 (p)>4 in candidate regions under selection

File name: Supplementary Data 8.

Description: Source data to Figure 4i

File name: Supplementary Data 9

Description: Source data to Figure 6

File name: Supplementary Data 10

Description: Genes in BayesR regions (canFam4)

File name: Supplementary Data 11

Description: Genes in XP-EHH regions (canFam4)

File name: Supplementary Data 12

Description: STRING result from BayesR genes (Homo Sapiens)

File name: Supplementary Data 13

Description: Results from STRING (Homo Sapiens) when including genes in both BayesR and XP-EHH regions

File name: Supplementary Data 14

Description: Genes from BayesR regions overlapping with human GWAS of related diseases

File name: Supplementary Data 15

Description: Genes from XP-EHH regions overlapping with human GWAS of related diseases

File name: Supplementary Data 16

Description: Alternative cutoff for effect variants in BayesR: top 50 effect variants per breed
