# Supplementary material for: Bayesian model and selection signature analyses reveal risk factors for canine atopic dermatitis
Source: Commun Biol. 2022 Dec 8;5:1348. doi: 10.1038/s42003-022-04279-8 (PMC9731970; doi:10.1038/s42003-022-04279-8)
Supplement: Supplementary file 20 — Reporting summary [file 42003_2022_4279_MOESM20_ESM.pdf]

nature portfolio

Corresponding author(s): Katarina Tengvall and Kerstin Lindblad-Toh

Last updated by author(s): Nov 11, 2022

Reporting Summary

Nature Portfolio wishes to improve the reproducibility of the work that we publish. This form provides structure for consistency and transparency in reporting. For further information on Nature Portfolio policies, see our Editorial Policies and the Editorial Policy Checklist.

Statistics

For all statistical analyses, confirm that the following items are present in the figure legend, table legend, main text, or Methods section.

n/a	Confirmed	
☐	☒	The exact sample size (*n*) for each experimental group/condition, given as a discrete number and unit of measurement
☒	☐	A statement on whether measurements were taken from distinct samples or whether the same sample was measured repeatedly
☐	☒	The statistical test(s) used AND whether they are one- or two-sided<br>*Only common tests should be described solely by name; describe more complex techniques in the Methods section.*
☐	☒	A description of all covariates tested
☐	☒	A description of any assumptions or corrections, such as tests of normality and adjustment for multiple comparisons
☐	☒	A full description of the statistical parameters including central tendency (e.g. means) or other basic estimates (e.g. regression coefficient) AND variation (e.g. standard deviation) or associated estimates of uncertainty (e.g. confidence intervals)
☐	☒	For null hypothesis testing, the test statistic (e.g. *F*, *t*, *r*) with confidence intervals, effect sizes, degrees of freedom and *P* value noted<br>*Give P values as exact values whenever suitable.*
☐	☒	For Bayesian analysis, information on the choice of priors and Markov chain Monte Carlo settings
☐	☒	For hierarchical and complex designs, identification of the appropriate level for tests and full reporting of outcomes
☐	☒	Estimates of effect sizes (e.g. Cohen's *d*, Pearson's *r*), indicating how they were calculated

Our web collection on statistics for biologists contains articles on many of the points above.

Software and code

Policy information about availability of computer code

Data collection	We gathered genotype data from the Illumina Canine HD BeadChip (173,662 SNPs) genotyping array (Illumina; San Diego, CA) generated from blood samples from dogs collected from privately owned dogs in collaboration with several veterinary clinics throughout Sweden (LR, GR, GSD, and WHWT), US (WHWT), and Switzerland (LR and GR). The latter included samples from dogs collected in Switzerland, Netherlands, Finland, Germany, and France. Saliva samples from the UK (LR and GR) were collected by owners of the dogs and posted to the research team, and genotyped by Negoen using the Illumina CanineHD 230K BeadChip (Illumina, San Diego, CA). Samples for each cohort were collected according to each country's regulation. Whole genome sequences from oxford nanopore technologies (ONT) were generated from four LR dogs (two cases and two controls).
Data analysis	We used plink (v. 1.90b4.9) and R (v. 4.9.2) with the following R-packages GENESIS (v. 2.24.0), GWASTools (v. 1.40.0), and SNPRelate (v. 1.28.0) to analyze the genotyped datasets. For imputation, we used SHAPEIT2 (r904) and IMPUTE2 (v. 2.3.2). For further analysis, we used fastPHASE (v. 1.4.8), rehh (v. 3.2.1), and BayesR (v. 1, update 01/04/2021). Furthermore we used Welch Two Sample T test (two-tailed) and boxplot (R package stats and graphics v. 4.1.2) and ANOVA (R package stats v. 4.1.2). For analyses of whole genome sequences from ONT we used: Guppy (v. 6.0.1), Sniffles (v.2.0.3), clair3, WhatsHap (v. 1.2.1), SnpEff (v 4.3.t), bedtools.

For manuscripts utilizing custom algorithms or software that are central to the research but not yet described in published literature, software must be made available to editors and reviewers. We strongly encourage code deposition in a community repository (e.g. GitHub). See the Nature Portfolio guidelines for submitting code & software for further information.

Data

Policy information about availability of data

All manuscripts must include a data availability statement. This statement should provide the following information, where applicable:

- Accession codes, unique identifiers, or web links for publicly available datasets
- A description of any restrictions on data availability
- For clinical datasets or third party data, please ensure that the statement adheres to our policy

SNP-chip genotypes (plink files: bed, bim, fam, pheno) after quality controls and relatedness filtering have been uploaded in SciLifeLab DOI:10.17044/scilifelab.21287139. The sequencing data of four Swedish LR were deposited to ENA with accession number for project: PRJEB55514, study: ERP140417, and samples: ERS10220104- ERS10220107.

Human research participants

Policy information about studies involving human research participants and Sex and Gender in Research.

Reporting on sex and gender	N/A
Population characteristics	N/A
Recruitment	N/A
Ethics oversight	N/A

Note that full information on the approval of the study protocol must also be provided in the manuscript.

Field-specific reporting

Please select the one below that is the best fit for your research. If you are not sure, read the appropriate sections before making your selection.

☒ Life sciences ☐ Behavioural & social sciences ☐ Ecological, evolutionary & environmental sciences

For a reference copy of the document with all sections, see nature.com/documents/nr-reporting-summary-flat.pdf

Life sciences study design

All studies must disclose on these points even when the disclosure is negative.

Sample size	For each breed we collected as many cases and controls as possible.
Data exclusions	Dogs lacking clear clinical diagnosis or healthy records for classifying as controls were removed. Samples lacking high quality genotypes were removed.
Replication	No replication was performed.
Randomization	Randomization was not applicable.
Blinding	No blinding.

Reporting for specific materials, systems and methods

We require information from authors about some types of materials, experimental systems and methods used in many studies. Here, indicate whether each material, system or method listed is relevant to your study. If you are not sure if a list item applies to your research, read the appropriate section before selecting a response.

Materials & experimental systems

n/a	Involved in the study
☒	☐ Antibodies
☒	☐ Eukaryotic cell lines
☒	☐ Palaeontology and archaeology
☐	☒ Animals and other organisms
☒	☐ Clinical data
☒	☐ Dual use research of concern

Methods

n/a	Involved in the study
☒	☐ ChIP-seq
☒	☐ Flow cytometry
☒	☐ MRI-based neuroimaging

Animals and other research organisms

Policy information about studies involving animals; ARRIVE guidelines recommended for reporting animal research, and Sex and Gender in Research

Laboratory animals	N/A. Pet dogs seeking health care were included in the study.
Wild animals	N/A
Reporting on sex	Registered sex was included in the information of each dog.
Field-collected samples	Blood or saliva samples collected from privately owned dogs.
Ethics oversight	All organizations performing sample collections had ethical approvals.

Note that full information on the approval of the study protocol must also be provided in the manuscript.
